# Temporal patterns of circulating cell-free DNA (cfDNA) in a newborn piglet model of perinatal asphyxia

**DOI:** 10.1371/journal.pone.0206601

**Published:** 2018-11-26

**Authors:** Sophia Manueldas, Torkil Benterud, Corina Silvia Rueegg, Håvard Tetlie Garberg, Marianne Ullestad Huun, Leonid Pankratov, Monica Åsegg-Atneosen, Rønnaug Solberg, Javier Escobar, Ola Didrik Saugstad, Lars Oliver Baumbusch

**Affiliations:** 1 Department of Pediatric Research, Division of Pediatric and Adolescent Medicine, Oslo University Hospital Rikshospitalet, Oslo, Norway; 2 University of Oslo, Oslo, Norway; 3 Oslo Centre for Biostatistics and Epidemiology, Department of Biostatistics, Institute of Basic Medical Sciences, University of Oslo, Oslo, Norway; 4 Department of Pediatrics, Vestfold Hospital Trust, Tønsberg, Norway; Indiana University, UNITED STATES

## Abstract

Perinatal asphyxia is a severe medical condition resulting from oxygen deficiency (hypoxia) at the time of birth, causing worldwide approximately 680,000 newborn deaths every year. Better prediction of severity of damages including early biomarkers is highly demanded. Elevated levels of circulating cell-free DNA (cfDNA) in blood have been reported for a range of different diseases and conditions, including cancer and prematurity. The objective of this study was to validate methods for assessing cfDNA in blood and cerebrospinal fluid (CSF) and to explore temporal variations in a piglet model of neonatal hypoxia-reoxygenation. Different cfDNA extraction methods in combination with cfDNA detection systems were tested, including a fluorescent assay using SYBR Gold and a qRT-PCR-based technique. Newborn piglets (n = 55) were exposed to hypoxia-reoxygenation, hypoxia-reoxygenation and hypothermia, or were part of the sham-operated control group. Blood was sampled at baseline and at post-intervention, further at 30, 270, and 570 minutes after the end of hypoxia. Applying the fluorescent method, cfDNA concentration in piglets exposed to hypoxia (n = 32) increased from 36.8±27.6 ng/ml prior to hypoxia to a peak level of 61.5±54.9 ng/ml after the intervention and deceased to 32.3±19.1 ng/ml at 570 minutes of reoxygenation, whereas the group of sham-operated control animals (n = 11) revealed a balanced cfDNA profile. Animals exposed to hypoxia and additionally treated with hypothermia (n = 12) expressed a cfDNA concentration of 54.4±16.9 ng/ml at baseline, 39.2±26.9 ng/ml at the end of hypoxia, and of 41.1±34.2 ng/ml at 570 minutes post-intervention. Concentrations of cfDNA in the CSF of piglets exposed to hypoxia revealed at post-intervention higher levels in comparison to the controls. However, these observations were only tendencies and not significant. In a first methodological proof-of-principle study exploring cfDNA using a piglet model of hypoxia-reoxygenation variations in the temporal patterns suggest that cfDNA might be an early indicator for damages caused by perinatal asphyxia.

## Introduction

Perinatal- or birth asphyxia is caused by insufficient oxygen dispersion to the fetus and/or neonate at the time of birth resulting in over 680,000 deaths of newborns every year [1]. The combination of hypoxia (insufficient oxygen supply), metabolic acidosis (increased acid levels), and ischemia (reduced blood perfusion) harms the vital organs of the neonates, including the heart, lungs, kidneys, and essentially the brain [2–4]. So far, hypothermia is the only established treatment for a significantly better neurologic outcome [5, 6]. Reliable and early diagnostic markers for the severity and overall outcome of perinatal asphyxia could substantially improve intervention strategies and treatment. A number of clinical and experimental animal studies indicate that perinatal asphyxia provokes cellular energy failure, generation of reactive oxygen species (ROS), formation of oxidative stress, and damages of cell-structures [7–10]. Previous studies have shown that biomarkers of ROS and reactive nitrogen species (RNS) can be measured shortly after hypoxic-ischemic injury [11]. These reactions are expected to trigger necrosis, apoptosis, and autophagy resulting in organ failures, like the clinical picture of hypoxic-ischemic encephalopathy [12]. To estimate oxidative stress reactions and damages in perinatal medicine, many different biomarkers have been investigated with unsatisfying results, including isoprostanes, isofuranes, gluthathione GSH/GSSG ratio, 8-hydroxy-2-deoxyguanosine, non protein-bound iron, superoxide dismutase, malondialdehyde, and S100B [13, 14].

The idea to investigate circulating cell-free DNA (cfDNA) using an asphyxia model of newborn piglets was inspired by a study reporting higher concentrations of circulating cfDNA in premature neonates [15] and by several publications dealing with the assessment of cfDNA in peripheral blood as potential novel biomarker for cancer [16–19]. An increasing number of studies points out that elevated levels of cfDNA are not cancer-specific, but reflect tissue pathology in various conditions and syndromes, including inflammatory diseases, trauma, stroke, hemodialysis, heavy exercise, sepsis, and pre-eclampsia [20–26]. Basic low values of cfDNA can even be detected in healthy individuals with higher levels in both, children and older individuals [27]. The amount of cfDNA in blood is highly variable ranging from a few to several thousand copies per milliliter blood, influenced by several factors like sex, weight, and general health [19]. The exact origin and release of cfDNA into the blood stream remains elucidative; but, apoptotic, necrotic, and lytic processes are likely to be involved [28, 29]. The expected turn-over time for cfDNA is rather short, suggesting a continuous flow of cfDNA into the blood stream [30], which is supported by the observation that an increase of cfDNA in healthy individuals can be determined minutes after incremental exercise [21]. Significant levels of cfDNA are further present in a range of other body fluids, including cerebrospinal fluid (CSF) [31, 32]. Fragments of free nuclear acids are typically 140 to 170 base pairs (bp) in length, and both, genomic and mitochondrial DNA are present as DNA fragments or nucleosome complexes [29, 33]. Multifarious extraction methods, quantification techniques, and applications for the assessment of cfDNA have been presented [34–37]; however, a golden standard is still missing. Further, all described methods have been tested and adjusted only for human samples. For obvious ethical reasons, novel intervention strategies and biomarkers for perinatal asphyxia are commonly tested in animal models. Piglets are favorable due to their anatomical, immunological, genetic, and physiological similarities to humans [38, 39]. However, temporal variations and patterns of cfDNA levels during different disease states have barely been measured and cfDNA has never been investigated in relation to perinatal asphyxia.

We hypothesized that perinatal asphyxia may provoke alterations in cfDNA level, which could be determined and possibly used as indicator for cellular and organ damages. To this end, we tested different extraction and measurement methods of cfDNA for a piglet model. Thus, the presented study is the first effort to estimate sequential changes of cfDNA levels in blood under the conditions of perinatal asphyxia including treatment with therapeutic hypothermia.

## Material and methods

### Aim

The aim of this study was to establish methods for cfDNA assessment and to investigate the temporal changes of cfDNA in blood for a clinically relevant piglet model of neonatal hypoxia-reoxygenation.

### Ethics approval

The Norwegian Council for Animal Research approved the experimental protocol (approval numbers 4630 for cohort I and 5723 for cohort II), animals were handled in accordance with the European Guidelines for the use of experimental animals by researchers certified by the Federation of European Laboratory Animals Science Association (FELASA).

It has been confirmed by the Regional Committees for Medical and Health Research Ethics (REC) that an oral consent is sufficient for taking a single, comparative human blood sample from a volunteer (one of the authors) and that further action or permission is not required.

### Animal samples

**Fig 1:**
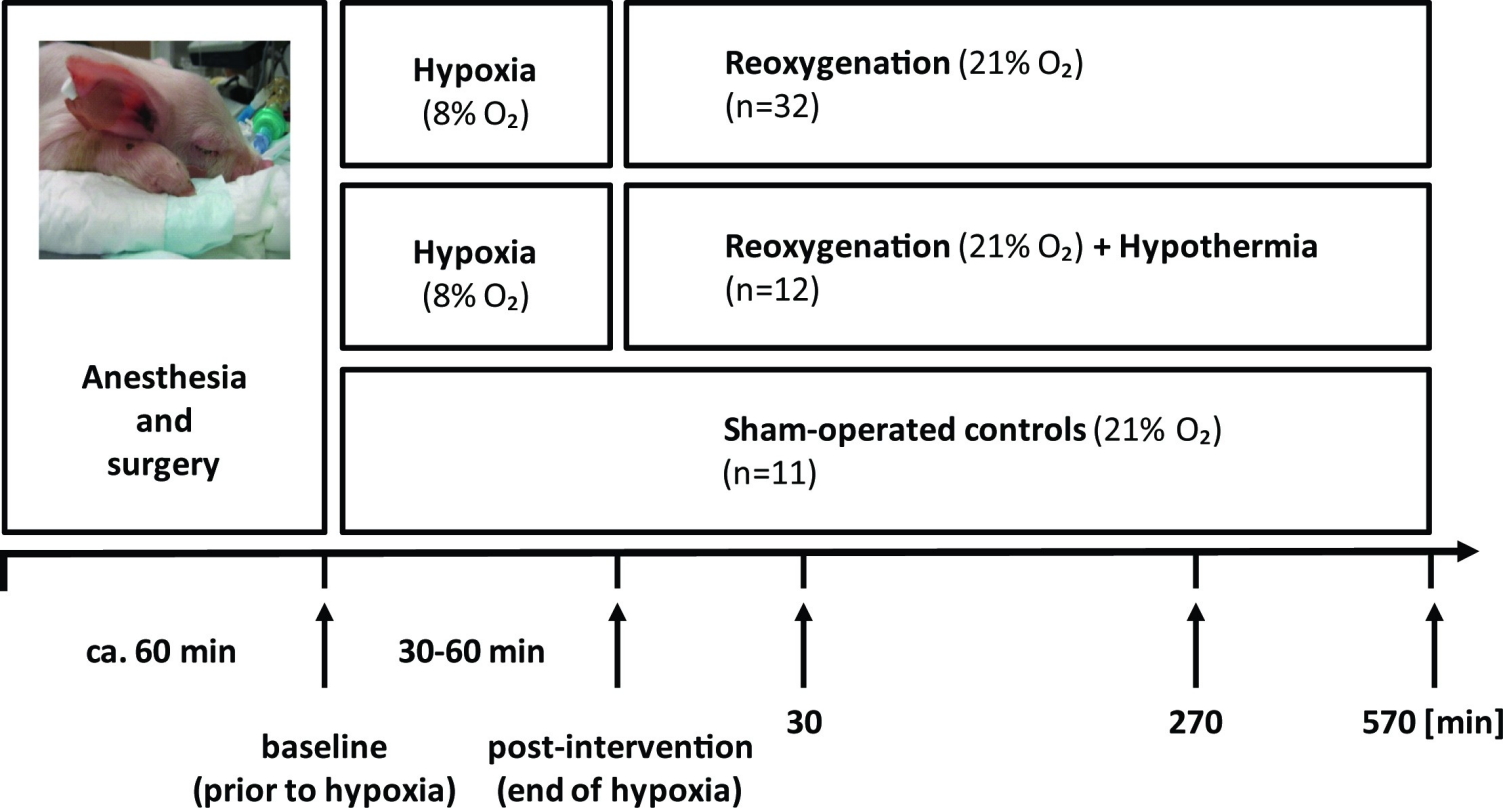
The perinatal asphyxia model of newborn piglets exposed to hypoxia-reoxygenation. A total of n = 55 newborn piglets were randomized into three study arms: hypoxia-reoxygenation, hypoxia-reoxygenation and hypothermia, or sham-operated controls. Perinatal asphyxia was mimicked by addition of CO_2_ aiming at a PaCO_2_ of 8.0–9.5 kPa until a BE of -20 mmol/l and/or mean blood pressure fell below 20 mm Hg. Blood samples for cfDNA determination were taken at baseline, one hour after stabilization and prior to hypoxia, then repeated after hypoxia, and further at 30, 270, and 570 minutes of reoxygenation. The CSF samples were collected at post-intervention, 570 minutes after the end of hypoxia.

A total of n = 55 newborn piglets were included in the study at an age of 12–36 hours and in good general condition. All newborn piglets were anesthetized, ventilated, and surgically prepared, as previously described in detail [40–42]. Briefly, after approximately one hour of stabilization the intervention groups (n = 32 and n = 12) were exposed to global hypoxia for approximately 30–60 minutes and followed for 9.5 hours (Fig 1). Perinatal asphyxia was mimicked by addition of CO_2_ aiming at a PaCO_2_ of 8.0–9.5 kPa. Piglets were resuscitated when base excess (BE) reached -20 mmol/l and/or mean blood pressure fell below 20 mm Hg [41]. For the hypothermia intervention group randomized newborn piglets (n = 12) were cooled to 35±0.5°C (recorded by a digital rectal thermometer) on a cooling mattress (Tecotherms TSmedd 200, TecCo, Halle, Germany) 30 minutes after the end of hypoxia, together with saline supplement [43]. Piglets in the sham-operated control group (n = 11) underwent the same surgery and anesthesia without intervention (Fig 1). Blood was sampled at baseline (prior to hypoxia) and at post-intervention (end of hypoxia), further at 30, 270, and 570 minutes after the end of hypoxia (= 570 minutes of post-intervention). For the blood samples approximately 2 ml of blood were centrifuged at 2.500 rpm for 10 minutes with a temperature set at 4°C. After centrifugation the supernatant, containing the plasma with cfDNA, was transferred to a new Eppendorf tube, snap frozen in liquid nitrogen, and stored at -70°C. CSF samples were taken at the end of the experiments, 570 minutes after the end of hypoxia (Fig 1). At 570 minutes of post-intervention all piglets were terminated by intravenous injection of 150 mg/kg pentobarbital. For comparison reasons, a sample blood of an adult pig was obtained from the Institute for Surgical Research, Oslo University Hospital, Oslo, Norway and a human blood sample taken from a voluntary healthy 46-year old male.

### Assessment of cfDNA

The cfDNA samples were extracted using the DNeasy Blood & Tissue Kit (DNeasy kit, Qiagen, Hilden, Germany) with a starting volume of 220 μl, 20 μl of proteinase K, and 200 μl elution buffer AE. Further cfDNA extraction kits and methods were tested, including NucleoSpin Plasma XS Kit (NucleoSpin kit, Macherey-Nagel, Duren, Germany) and Wizard Genomic DNA Purification Kit (Wizard kit, Promega, Madison, USA) (S1 File). Plasma samples from piglets were spiked with 100 ng/μl standard for a recovery test using the DNeasy kit. Additionally, some plasma samples were treated with Dnase (Bio-Rad, USA) to achieve zero control samples. Three different samples with parallels were analyzed (S1 File).

The fluorescent assay for cfDNA was based on previously reported methods [44, 45]. We tested SYBR Gold due to its description of being extremely high sensitive for single- or double-stranded RNA or DNA [44, 45]. Briefly, SYBR Gold Nucleic Acid Gel Stain (Invitrogen, Paisley, UK) was diluted 1:1000 in dimethyl sulphoxide (DMSO) (Sigma-Aldrich, Missouri, USA) and kept in the dark. For measurements, aliquots were diluted 1:8 in phosphate-buffered saline (PBS). 10 μl of sample solution were applied to a black Nunc MicroWell 96-well Microplate (Roskilde, Denmark) and 40 μl of diluted SYBR Gold was added to each sample, resulting in a final concentration of 1:10.000. The fluorescence was immediately measured at an emission wavelength of 535 nm and an excitation wavelength of 485 nm using a Victor TM X3 (Perkin Elmer, Waltham, USA).

Different sources of DNA were tested to prepare standard curves for the fluorescent methods: deoxyribonucleic acid, low molecular weight from salmon sperm (Sigma-Aldrich, Missouri, USA), Human Genomic DNA: Male (Promega, Madison, USA), and Control Genomic DNA: Porcine Female from normal tissue (Amsbio, Abingdon, UK) (S1 Fig). All standards were diluted in PBS to different concentrations and photometrically measured with SYBR Gold (S1 Fig).

The size distribution of cfDNA is rather scattered [46], convincing us to perform fragmentation test for our DNA standards. The used porcine DNA has a higher fragment size and consequently different procedures, like fragmentation via UV or restriction enzyme digestion, were tested to adjust the length of the standard DNA to naturally cfDNA sizes (S2 Fig). This was done in two different ways, 1. fragmentation of DNA into small fragments by incubation of porcine stock in UV-bath for 5 minutes and then followed by a serial dilution or 2. fragmentation of DNA with restriction enzyme *HhaI*. The reaction mix contained 3.5 μl of 0.53 μg/μl porcine DNA and 1 μl smart cut buffer, 5 U *HhaI* enzyme and MQ water to a final volume of 10 μl. The DNA was digested for 1 hour at 37°C and inactivated in 10 minutes at 65°C. After fragmentation, the stock was diluted with PBS to various concentrations. Nevertheless, the results shown here exhibited that the different fragment sizes did not influence the results.

To measure the quantity of cfDNA by qRT-PCR technique, multiple target genes have been applied, including GAPDH, hTERT, β-globulin, or β-actin [19]. We investigated several suggested primers for β-actin from studies on humans [47–49]; however, many of these primers matched to multiple sites, to pseudogenes, and/or poorly to the target site. Further investigations were inspired by a comprehensive study on expression reference genes on piglets [50]. In this study, the presented target for hydroxymethylbilane synthese (HMBS), a single target gene, seemed promising and consequently, primers were designed for the location. The signals for the first HMBS primers were too low convincing us to design novel HMBS primers with a smaller product, in order to catch also smaller cfDNA fragments. Next, we tested β-globulin, which has been preferred in many studies [23, 44, 45, 51, 52]. The gene for phosphomannomutase 1 (PMM1) has been described as a stable endogenous control for expression studies in humans [53]. Based on the *sus scrofa* (*ss*) sequence (NC 010447), we constructed novel primers for HMBS, β-globulin, and PMM1 using the Primer3Plus program [54] with settings following the recommendations of Applied Biosystems (Tm 58–60°C, GC-content 40–70%, primer length 18–25 bp, < 2°C difference in Tm between primers, maximum of 2/5 G or C at the 3’ end, < 4 contigious G,C,A,T, and 50–150 bp in length). Sequences, annealing temperature, and product size are listed in Table 1. The qRT-PCR mixture contained 5 μl DNA template or DNA standards (6.25–1250 ng/ml), 2 μl of each primer (20 μmol/l), 12 μl SYBR mix, and MQ-water to a final volume of 25 μl. The qRT-PCR program used for quantification of cfDNA was: an initial activation step of 50°C for 2 min and 95°C for 10 min, followed by 40 cycles of denaturation at 95°C for 15 s and annealing and elongation at 59°C for 60 s, finalized by a dissociation step at 95°C for 15 s. Samples were all run in parallels. The reaction was carried out in Applied Biosystems Viia7 qRT-PCR (Life technologies, Foster City, USA).

**Table 1:** Novel designed primers for the qRT-PCR method.

**HMBS (Hydroxymethylbilane synthase)—long**			
Forward primer	FP HMBS-ss_6083	5’-gcttcagagaaagttcccaca-3’	Tm: 59.5°C
Reverse primer	RP HMBS-ss_6183	5’-ggccttctggacctcattt-3’	Tm: 59.1°C
		Product:	100 bp
**HMBS (Hydroxymethylbilane synthase)—short**			
Forward primer	FP HMBS-ss_6220	5’-gtagaccatggatggcagtg-3’	Tm: 59.0°C
Reverse primer	RP HMBS-ss_6576	5’-ttacgaatggatggatggaa-3’	Tm: 58.8°C
		Product:	56 bp
**β-globulin**			
Forward primer	FP β-globulin-ss_140	5’-gcaagctgctggttgtctac-3’	Tm: 58.7°C
Reverse primer	RP β-globulin-ss_273	5’-gtcactgaaggactggagca-3’	Tm: 59.0°C
		Product:	134 bp
**PMM1 (Phosphomanno-mutase 1)**			
Forward primer	FP PMM1-ss_control	5’-gagattccctggagctgtgt-3’	Tm: 59.3°C
Reverse primer	RP PMM1-ss_N-control	5’-attctgtccgctttgttcct-3’	Tm: 58.8°C
		Product:	153 bp

### Statistics

Statistical analysis was performed using STATA Statistical Software version 14 (StataCorp LP, College Station, Texas, USA). We present numbers and proportions as well as means and standard deviations to describe the characteristics of the piglets in the project at baseline and over time.

To explore the methods used to assess cfDNA, we draw boxplots displaying median, interquartile range and range to present the following: 1. Comparison of cfDNA blood concentration by fluorescence method in adult human, adult pig, and newborn piglets, 2. cfDNA blood concentration at baseline and post-intervention comparing fluorescence method and qRT-PCR method, overall and stratified by study arm, and 3. comparison of CSF concentration at study end comparing the fluorescence method and qRT-PCR method, overall and stratified by study arm. We calculated p-values for all of these comparisons using chi-squared test statistics on a nonparametric K-samples test on the equality of medians. Spearman rank-correlation coefficients were calculated between the fluorescent assay and the qRT-PCR technique to assess the agreement between the two methods (for blood concentration at baseline and post-intervention and for CSF concentration at post-intervention).

To investigate the sensitivity of cfDNA as a marker of hypoxia, we described and modeled the cfDNA levels by study arm and over time, using a mixed model for repeated measures. We performed a Receiver Operator Characteristics (ROC) curve to assess the sensitivity and specificity of cfDNA 30 minutes post-intervention to discriminate between piglets with and without hypoxia.

## Results

### Testing methods for extraction and measurement of cfDNA

To optimize cfDNA extraction, the DNeasy Blood & Tissue Kit from Qiagen, NucleoSpin Plasma XS Kit from Macherey-Nagel, and Wizard Genomic DNA Purification Kit from Promega were tested and compared in regards to handling time, purity, starting volume, and yield (S1 File).

Two common techniques were validated to assess the amount of cfDNA extracted from the samples: a fluorescent-based assay and a qRT-PCR method. For the fluorescent-based assay, salmon, human, or porcine DNA were chosen as basis for the standard curve and both, the human and the porcine standard curves expressed linearity up to 1250 ng/μl; however, the porcine resulted in a higher total range, above the human, and was used for the following experiments (S1 Fig). So far, cfDNA has only been measured in human samples. To get a reference value, we determined the amount of cfDNA in human blood plasma using the fluorescent assay with SYBR Gold. The human sample revealed a mean concentration of about 35 ng/ml versus 20 ng/ml in an adult pig (S3 Fig). In contrast, the concentrations of cfDNA in the newborn piglets used for our study were twice as high as in an adult pig, 20.1±1.1 ng/ml versus 37.3±18.3 ng/ml, respectively (S3 Fig).

### Temporal changes of cfDNA levels in a piglet model of asphyxia

**Fig 2:**
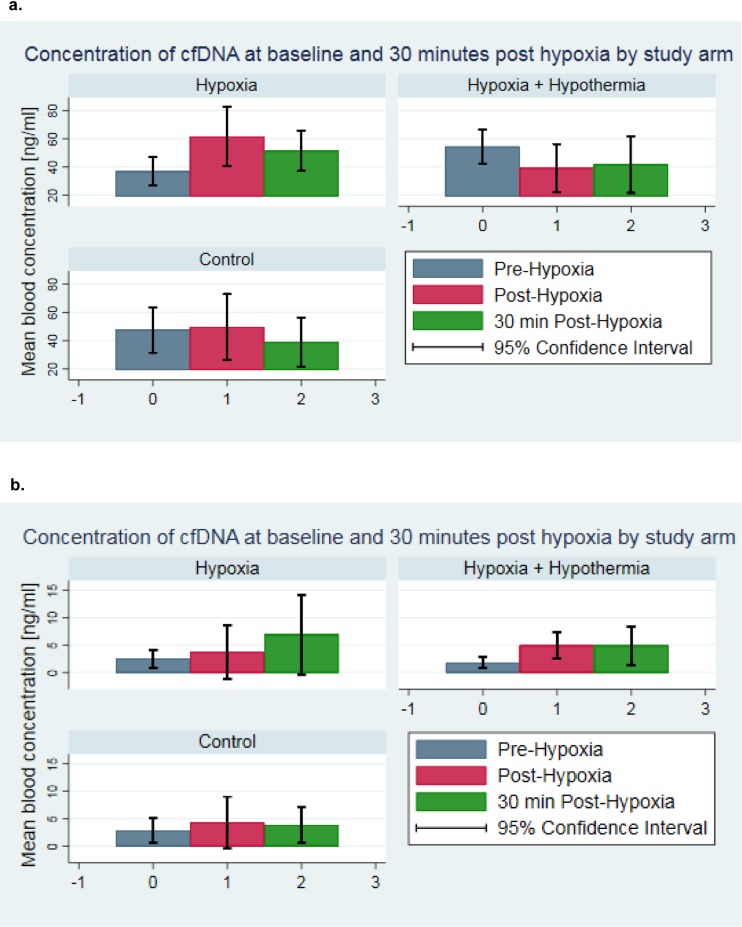
Changes in the concentrations of cfDNA in plasma of newborn piglets exposed to hypoxia, stratified by study arm. The amount of cfDNA in blood plasma of newborn piglets exposed to hypoxia-reoxygenation was determined at baseline (prior to hypoxia), post-intervention (end of hypoxia), and at 30 minutes after the end of hypoxia. a. Mean cfDNA blood concentration and 95% confidence intervals stratified by study arm were determined using the fluorescence-based assay. The p-values from paired t-tests comparing baseline versus end of hypoxia values were p = 0.971 for controls, p = 0.009 for animals of the hypoxia group, and p = 0.193 for the hypoxia and hypothermia group, respectively. The p-values comparing baseline versus 30 minutes post-intervention were p = 0.245 for the control-, p = 0.032 for the hypoxia-, and, p = 0.131 for hypoxia and hypothermia group, respectively. There were statistically significant differences between the study arms comparing the difference from baseline to end of intervention (p = 0.038) and the difference from baseline to 30 minutes post-hypoxia (p = 0.025). b. Mean cfDNA blood concentration and 95% confidence intervals prior to hypoxia, at the end of hypoxia and 30 minutes after the end of intervention for the qRT-PCR method using the novel designed β-globulin primers, stratified by study arm. The p-values from paired t-tests comparing baseline versus end of hypoxia were p = 0.393 for the control-, p = 0.452 for the hypoxia-, and, p = 0.010 for the hypoxia and hypothermia group, respectively. The p-values comparing prior to intervention versus 30 minutes after the end of hypoxia were p = 0.421 for the sham-control animals group, p = 0.212 for the hypoxia group, and p = 0.071 for hypoxia and hypothermia group, respectively. There was no statistically significant difference between the study arms comparing the difference from baseline to end of hypoxia (p = 0.637) and the difference from baseline to 30 minutes after the end of intervention (p = 0.674). The cfDNA concentrations of additional 14 piglets were measured prior to surgery applying the fluorescence method, their mean blood concentration was 25.4 ng/ml (SD = 19.6; 95%CI 14.11–36.72).

**Fig 3:**
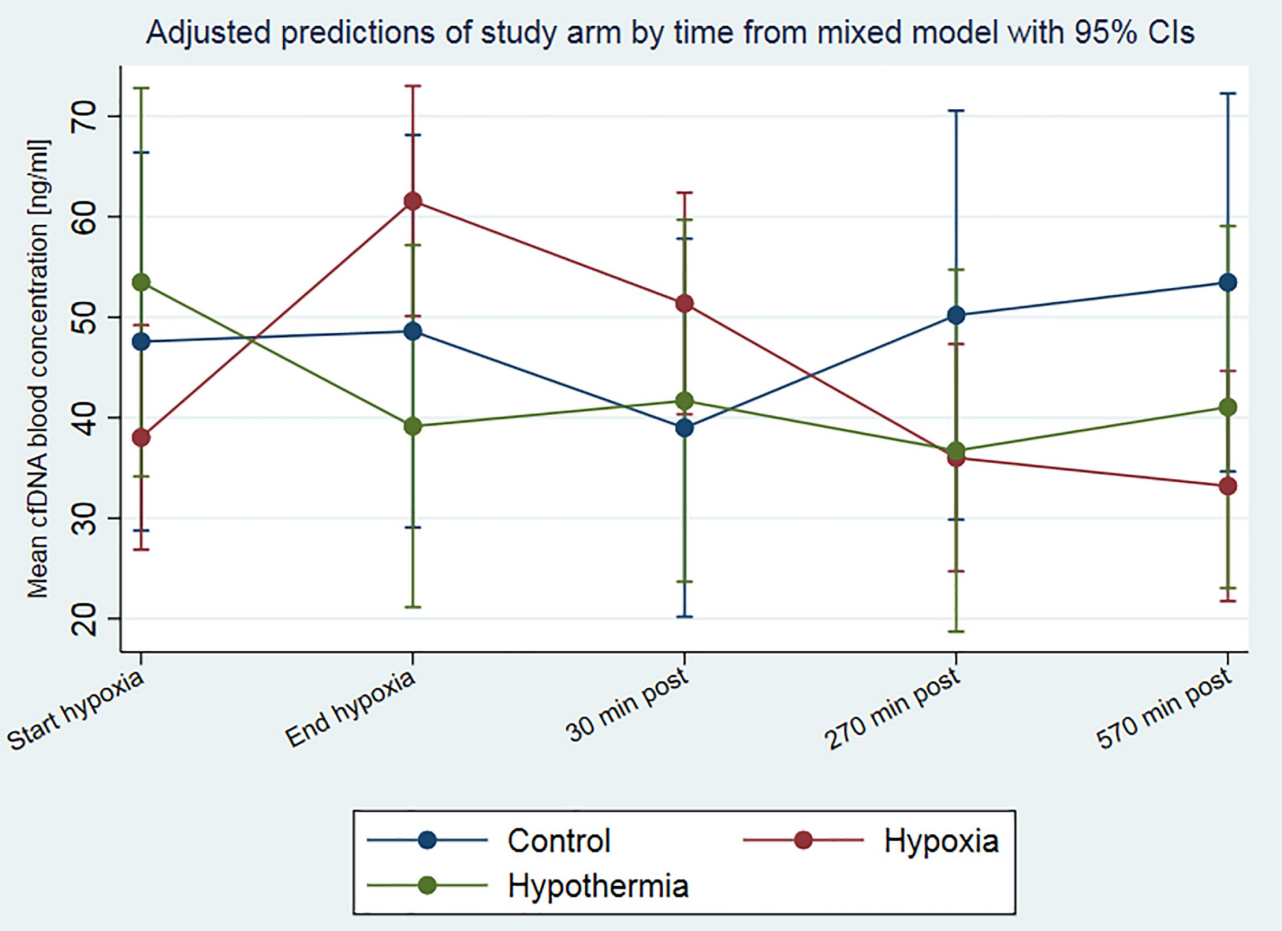
Predicted mixed model of temporal pattern of cfDNA in plasma of newborn piglets, stratified by study arm. Concentrations of cfDNA were measured in newborn piglets exposed to hypoxia-reoxygenation, hypoxia-reoxygenation in combination with hypothermia, or in sham-operated control animals. The fluorescence method was applied for cfDNA determination. The mean cfDNA blood concentration and 95% confidence intervals from the fluorescence method for the different time points are illustrated and stratified by study arm. Mean blood concentration and 95% confidence intervals are predicted from an univariable linear mixed effects model with random intercept. There were no overall statistically significant effects of study arm (p = 0.838) and time (p = 0.570) on the cfDNA concentration, but there was a significant interaction between study arm and time (p = 0.018).

**Fig 4:**
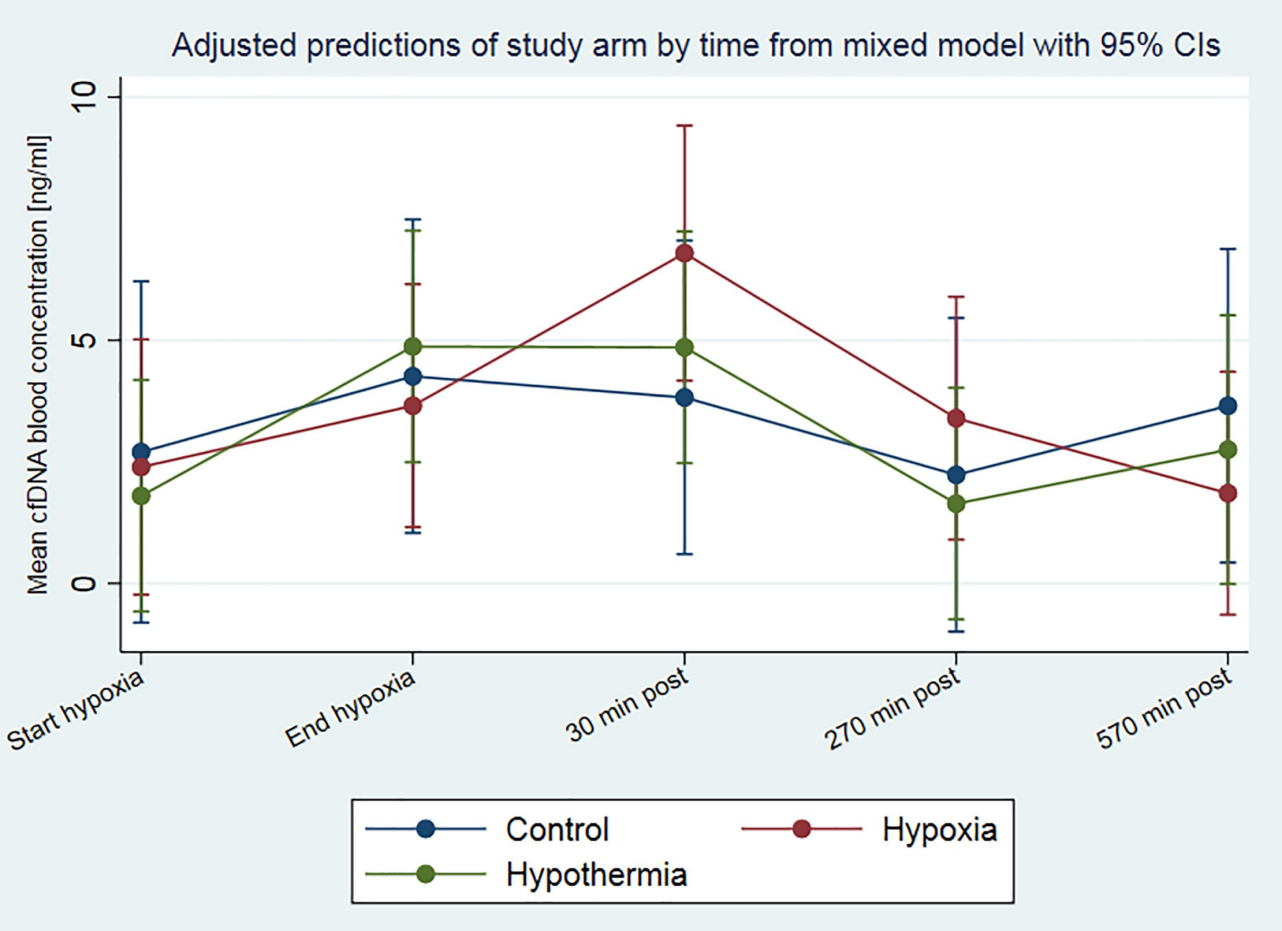
Predicted mixed model of mean cfDNA blood concentration of newborn piglets exposed to hypoxia-reoxygenation measured by qRT-PCR and stratified by study arm. The mean cfDNA blood concentration and 95% confidence intervals from the qRT-PCR method are illustrated for the different time points and stratified by study arm. Mean blood concentration and 95% confidence intervals are predicted from an univariable linear mixed effects model with random intercept. There were no overall statistically significant effects of study arm on the cfDNA concentration (p = 0.9143) or the interaction between study arm and time (p = 0.736). There was a significant effect of time on the cfDNA blood concentration (p = 0.022).

The physiological variables exhibited no significant differences at baseline between the hypoxia versus sham-operated control groups, for the parameters weight, pO_2_, pCO_2_, lactate, or BE [40, 42]. Applying the fluorescence method for cfDNA estimation, piglets exposed to hypoxia exhibited a mean cfDNA level of 36.8±27.6 ng/ml at baseline (start of hypoxia) and an evident peak of 61.5±54.9 ng/ml at the end of hypoxia (Table 2 and Fig 2). The amount of cfDNA in blood plasma decreased continuously during the time of reoxygenation from 51.4±39.5 ng/ml at 30 minutes to 36.3±24.0 ng/ml at 270 minutes and finally 32.3±19.1 ng/ml after 570 minutes of reoxygenation at the end of the experiment (Table 2, Fig 3 and Fig 4). The sham-control animals revealed a balanced cfDNA profile over time, at baseline and the time of the end of the hypoxia it was at 47.6±23.9 ng/ml and 49.7±32.5 ng/ml, respectively (Table 2 and Fig 2). After 30 minutes of post-intervention the cfDNA levels first dropped to 39.0±25.8 ng/ml before they slightly increased to 50.9±24.9 ng/ml 53.5±38.1 ng/ml at 270 minutes and 570 minutes, respectively (Table 2, Fig 3 and Fig 4). The group of hypoxia and hypothermia treated animals revealed the highest cfDNA baseline values of all groups with 54.4±16.9 ng/ml, dropping to 39.2±26.9 ng/ml at the end of hypoxia and remained more or less stable with 41.7±31.5 ng/ml, 36.7±17.9 ng/ml, and 41.1±34.2 ng/ml at 30, 270, and 570 minutes of post-intervention (Table 2, Fig 3 and Fig 4). The visual differences between these groups were only tendencies and not significant.

**Table 2:** Characteristics and mean cfDNA concentrations in ng/ml at different time points of the study for the included piglets.

			Hypoxia		Hypoxia & hypo-thermia		Controls		
			(n = 32^a^)		(n = 12)		(n = 11^b^)		
	**Tissue**	**Method**	**N**		**N**		**N**		
*Material*									
	Blood		20		12		6		
	Blood & CSF		12		0		5		
*Method*									
	Blood	Fluorescence	32		12		11		
	Blood	qRT-PCR	10		11		6		
	CSF	Fluorescence	12		0		5		
	CSF	qRT-PCR	12		0		5		
**Time point**	**Tissue**	**Method**	**Meanng/ml**	**SD ng/ml**	**Meanng/ml**	**SD ng/ml**	**Meanng/ml**	**SD ng/ml**	**p-value**
Baseline	Blood	Fluorescence	36.8	27.6	54.4	16.9	47.6	23.9	0.128
Baseline	Blood	qRT-PCR	2.5	2.2	1.8	1.5	2.8	1.8	0.530
Post-intervention	Blood	Fluorescence	61.5	54.9	39.2	26.9	49.7	32.5	0.358
Post-intervention	Blood	qRT-PCR	3.7	6.9	4.9	3.6	4.3	4.5	0.868
30 minutes post-intervention	Blood	Fluorescence	51.4	39.5	41.7	31.5	39.0	25.8	0.525
30 minutes post-intervention	Blood	qRT-PCR	6.9	9.4	4.9	5.2	3.8	3.1	0.664
270 minutes post-intervention	Blood	Fluorescence	36.3	24.0	36.7	17.9	50.9	24.9	0.239
270 minutes post-intervention	Blood	qRT-PCR	3.4	4.1	1.6	1.3	2.2	1.3	0.337
570 minutes post-intervention	Blood	Fluorescence	32.3	19.1	41.1	34.2	53.5	38.1	0.104
570 minutes post-intervention	Blood	qRT-PCR	1.9	1.7	2.6	2.8	3.7	4.3	0.496
570 minutes post-intervention	CSF	Fluorescence	107.2	242.8	n.a.	n.a.	82.7	113.8	0.835
570 minutes post-intervention	CSF	qRT-PCR	98.0	225.0	n.a.	n.a.	89.0	109.6	0.934

^a^ Two piglets were excluded, one due to medical and one due to technical reasons.

^b^ One piglet of the control group was excluded due to medical reasons.

Abbreviations for Table 2: cfDNA—cell-free DNA; conc.—concentration; CSF—cerebrospinal fluid; N—number; n. a.—not applicable; qRT-PCR—quantitative real-time polymerase chain reaction; SD—standard deviation.

The qRT-PCR method using β-globulin primers resulted in lower levels of cfDNA (Fig 2A versus 2B). The hypoxic piglets showed a mean concentration of cfDNA of 2.5±2.2 ng/ml at baseline and 3.7±6.9 ng/ml at post-intervention versus 2.8±1.8 ng/ml and 4.3±4.5 ng/ml for the sham-control group, respectively (Table 2, Fig 2B). A comparative study of the fluorometric versus qRT-PCR method in our samples revealed only minor changes with slightly increased values with SYBR Gold (Fig 2A versus 2B) and a detection limit of about 1 ng/ml. To evaluate the consistency between the fluorescent assay and the qRT-PCR technique, correlation analyses were performed. At baseline, the spearman’s rank-correlations resulted in rho = 0.05 and at post-intervention in rho = -0.21 (S4 Fig). For cfDNA samples from CSF, the comparison of the two methods revealed a spearman’s rank-correlation of rho = 0.77 (S5 Fig).

### Sensitivity and specificity of cfDNA to detect hypoxia

**Fig 5:**
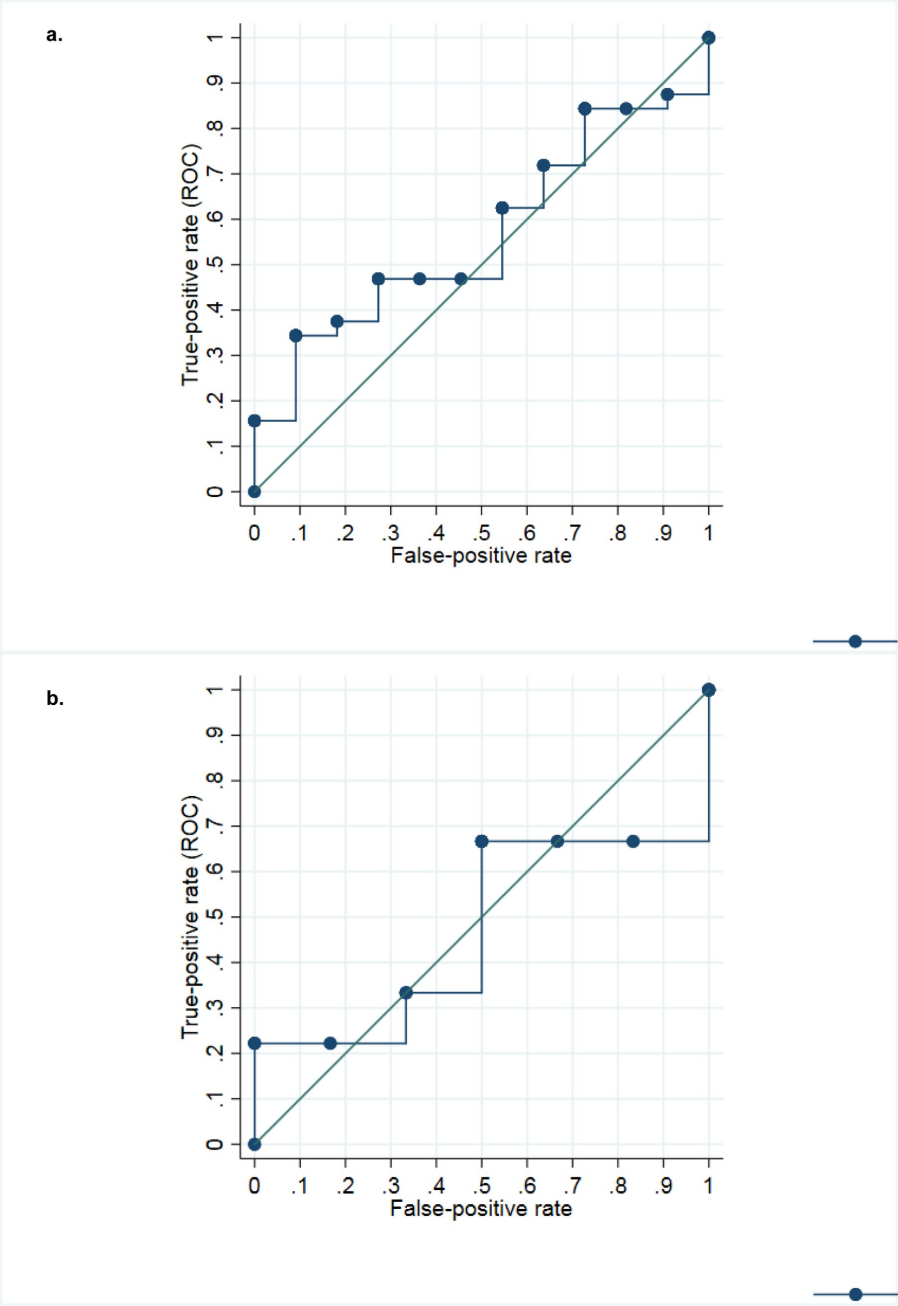
ROC curves for the discrimination between hypoxia and control piglets at time point 30 minutes post-intervention by cfDNA plasma levels. Receiver operating characteristic (ROC) curves are illustrated for a. cfDNA levels determined by fluorescence method (n = 43) and b. cfDNA levels measured using the qRT-PCR assay (n = 15) for the discrimination of piglets who did or did not undergo hypoxia intervention. The study arm of the group of piglets exposed to hypoxia and hypothermia was excluded from this analysis. The cfDNA by fluorescence method resulted in an area under the curve of 56%, the qRT-PCR method of 46%, respectively (p = 0.143).

The receiver operating characteristic (ROC) curves for distinguishing piglets with and without hypoxia 30 minutes post-intervention showed an area under the curve (AUC) of 56% (95% CI 37%-75%) for the fluorescence method and an AUC of 46% (95%CI 14%-78%) for the qRT-PCR method (Fig 5). The sensitivity and specificity reached by the fluorescence method at the optimal cut-off of 46.0 ng/ml were 47% and 73%, respectively. For the qRT-PCR method, the optimal cut-off was 1.8 ng/ml with a sensitivity of 67% and specificity of 50%.

### The concentrations of cfDNA in CSF

Piglets treated with hypoxia-reoxygenation showed a mean concentration of 107.2±242.8 ng/ml versus 82.7±113.8 ng/ml for the sham-operated control group applying the photometric method and 98.0±225.0 ng/ml versus 89.0±109.9 ng/ml, respectively, applying the qRT-PCR technique (Table 2). Notably, four piglets with symptoms of additional extreme stress reactions, including long stabilization time, blood in the lungs or swelling, and edema of the brain, revealed extremely high cfDNA values, 3–18 times above the average values of the other piglets.

## Discussion

### Validation of methods for extraction and quantification of cfDNA

We have tested and established methods to quantify cfDNA in piglets. Robust and reliable methods for the isolation and quantification of cfDNA are crucial for accurate assessment of the amount of cfDNA in a sample. Various techniques have been presented previously, including phenol-chloroform extraction, column-based kits, or magnetic bead-based systems, exploiting the principal differences in DNA extraction based on organic solvents, selective silica-gel membranes, or magnetic beads [19, 55]. All of these methods show high variability for the reproducibility, sensitivity, efficiency, purity, and overall yield, affecting the quality and reliability of the quantification results [19, 55]. A number of previous studies have validated different combinations of the diverse DNA isolation techniques; however, with inconclusive results and all were purely tested on human samples [34–37, 56, 57].

Various different techniques have been applied to investigate the concentration of cfDNA in blood and other body fluid samples including Nanodrop ND-1000, analysis of agarose or polyacrylamide gels, fluorometry with SYBR Green, SYBR Gold, Hoechst dye 33342 or PicoGreen, capillary zone electrophoresis, qRT-PCR, or digital PCR [19, 55]. Fluorometric- or qRT-PCR-based methods are the most frequently used techniques due to their sensitivity, handling, and accuracy [23, 27, 32, 35, 37, 45, 49, 58–61] and accordingly, we examined both major techniques for blood plasma and CSF samples from piglets. For fluorometric assays of cfDNA detection, chromophores like SYBR Green, SYBR Gold, Hoechst dye 33342, or PicoGreen may be used; we preferred the flurophore SYBR Gold in this study, based on its extremely high sensitivity for single- or double-stranded RNA or DNA [44, 45]. For the quantification of cfDNA involving the qRT-PCR method, a large selection of targets has been presented, e.g. ALU sequences, SRY, GAPDH, ERV, hTERT, RPPH1, POLR2, β-globulin, and β-actin genes, with high variations concerning sensibility and reproducibility [23, 34, 35, 44, 45, 48, 49, 59, 62–65]. However, for piglet studies, there was a lack of applicable cfDNA target primers and thus novel cfDNA target sites primers for piglets were designed. A comparison of the fluorescent-based assay versus the qRT-PCR method performing correlation analysis revealed minor changes with to some extent higher values for the SYBR Gold based assay. Consequently, the fluorescence assay was preferably performed, due to its simple and robust features, exhibiting a detection limit of about 1 ng/ml. Fluorometric assays are often quoted as more non-specific methods resulting in general higher levels, since chromophores bind to all sorts of nucleic acids present in the circulation, including genomic or mitochondrial DNA and coding- or non-coding RNAs. A common problem, which is ignored in many studies, is the possible contamination of genomic DNA, which might be a source for higher values. On the other hand, the more sensitive and specific qRT-PCR technique misses shorter DNA fragments and depends on the assumption that the target gene is representative for the entire amount of cfDNA. For future studies, it would be interesting to choose several different target genes to control for target gene-selective cfDNA quantification. Further, qRT-PCR using Taq-Man probes has been presented to increase the sensitivity of the cfDNA detection [47]. The system may further be improved by other targets or by comparing nuclear versus mitochondrial cfDNA [61, 66, 67].

The amount of cfDNA in human blood plasma was determined with a mean concentration of about 30 ng/ml, which corresponds to values previous presented in the literature [55, 68]. Reference values for cfDNA in the blood plasma of healthy individuals, presented in individual or meta-analysis studies, show a wide range of about 2.5 to 128 ng/ml and with an average of 15 ng/ml and about 30 to 100 ng/ml in serum; however, with generally high variations and many measurement uncertainties [19, 55]. In contrast to humans, adult pigs show a lower quantity of cfDNA of about 20 ng/ml and newborn piglets exhibit nearly twice the amount of cfDNA in comparison to adult pigs. This variation is in line with other observations of age- and sex-dependent distributions of the amounts of cfDNA in human serum, with higher amounts for young males, and females and lowest contents for males and females aged 40–50 years [27].

### The level of cfDNA in CSF

Circulating nucleic acids are not only released to the serum and plasma of blood, they also occur in a wide range of other body fluids, including urine, CSF, saliva, breast milk, and feces [31, 32]. This observation is making it reasonable to believe that similar mechanisms of cell damage and the exchange of nucleic acids fragments are distributed throughout the body. We demonstrated that it is possible to extract cfDNA from the CSF of piglets. The control group exhibited cfDNA levels in the CSF similar to those in blood plasma opening for speculation of either parallel originating of cfDNA in CSF and blood or an exchange of circulating nucleic fragments across the blood-brain barrier.

### Temporal variations in cfDNA using a piglet model of asphyxia

To the best of our knowledge, this is the first study investigating temporal variations in the amount of cfDNA using a piglet model of perinatal asphyxia. Temporal variations and patterns of cfDNA levels during different disease states have barely been measured and cfDNA has never been investigated in relation to perinatal asphyxia. We were able to identify rapid changes in the levels of cfDNA in piglets exposed to hypoxia with a peak after 30 minutes with the fluorescence method, whereas the sham-control animals revealed a balanced cfDNA profile. A response time of 30 minutes is a fast response and similar temporal changes could be shown for the miRNA miR-374a and miR-210 [43]. This coincides with clinical observations and experimental studies of damaged cell- and DNA structures following neonatal asphyxia [7–9]. Levels normalized quickly to baseline after reoxygenation suggesting a rapid clearance and turn over from plasma, which is similar to previous observations of a rapid turn-over of cfDNA in plasma, for example in healthy individual after heavy exercise [21]. Nevertheless, the exact biological origins and turn-over of cfDNA remain unclear. Therapeutic hypothermia is still the only clinically implemented treatment of hypoxic-ischemic encephalopathy and results in improved neurologic outcome and reduced mortality [5, 6, 69]. The baseline level of cfDNA in the hypothermia group of our piglet study was higher in comparison to the other groups. The cfDNA level immediately decreased (without a peak at the end of hypoxia) and remained low similar to the amounts of the sham-operated control group. Besides providing a proof of principle for measuring cfDNA in newborn piglets in an asphyxia model, we are aware that the observed changes are not significant and one may argue that the observed differences are only due to variations caused by the relatively low number of tested animals and of biological variations. The observation that the starting amount of cfDNA in the hypothermic piglets was above the others may support the idea that anesthesia and surgery are more dramatic events than the hypoxia itself. The present study provides novel clues about the temporal patterns of cfDNA release during a model of perinatal asphyxia. Further studies including a higher number of animals may provide more conclusive results.

### Demands for potential biomarkers for perinatal asphyxia

Reliable diagnostic biomarkers for the early prediction of the severity and outcome of perinatal asphyxia are highly demanded to guide clinical intervention. Several biomarkers have been examined to estimate hypoxic-ischemic injury in plasma, serum, and CSF, like nerve tissue markers, oxidative stress markers, calcium binding proteins, vasoactive agents, inflammatory mediators, and metabolic markers [70–72]. Preferable biomarkers should be simple with precise feasibility, high reliability, sensitivity, specificity, low cost, non-invasive, have the possibility for longitudinal monitoring, and the results should be available within 120 minutes; however, studying next generation biomarkers for brain injury in perinatal medicine none of the tested markers full-filled all of the requested criteria [72]. For obvious ethical reasons, the molecular mechanisms and novel intervention strategies for perinatal asphyxia can only be tested in animal models. Newborn piglets show high similarities to human neonates born in the gestational week 36–38 in relation to body weight and brain development [73, 74]. Consequently, piglets have been used frequently for studies of oxidative stress conditions in newborns [40, 75, 76]. The observation that cfDNA levels are increased under various conditions suggests that release of free nuclear acids fragments may reflect a general oxidative stress reaction. The increase of the amounts of cfDNA was found immediately after hypoxia, during what is called the "therapeutic window" following hypoxia and thus, may the increase in cfDNA have prognostic potential. However, the ROC curves showed no ability of cfDNA in distinguishing piglets with or without hypoxia 30 minutes post-intervention.

The level of cfDNA has not been previously investigated in the context of asphyxia and using a newborn piglet model. The strength of our study is the standardized protocols and conditions of sample collection and handling, both for the animal experiments and in the wet-lab. The presented initial studies were performed as proof-of-principle for the individual methods. Further studies have to proof the role of cfDNA as assessment for organ damage.

### Limitation of the study and outlook

Some authors state that the role of cfDNA as novel biomarker, at least for cancer, might be overrated [55]. Nevertheless, the assessment of cfDNA seems to be a good indicator for oxidative stress injuries, providing that the methodological challenges are solved. Numerous different techniques have been applied to investigate the concentrations of cfDNA in blood and other body fluids. A general problem for all cfDNA estimations is that there is no golden standard how to extract and quantify cfDNA, making the comparison to previous studies difficult, including the assessment of validity and sensitivity of methods applied by us. In addition, all DNA extraction methods share the disadvantage that some fragments are lost in the extraction process; thus, directly assessment of cfDNA without prior DNA extraction might be a better choice, as suggested by some authors [45, 77]. Our comparison of a fluorescent assay to qRT-PCR techniques revealed high similarity for cfDNA quantities in CSF and some difference for the amount of cfDNA in blood. This might be explained by the diverse sampling time (pre- and post-intervention for blood and 570 min after post-intervention for CSF), the higher amount of unspecific DNA-catching and -degrading agents in blood, and/or the potentially higher plausibility for oxidative stress damages in the brain.

Further, little is known about cfDNA in CSF and no reference values are available. One of the major challenges for this study was the generally low amount of cfDNA making the analysis more sensitive to variations. To increase the number of animals in each group may help to reduce the large standard deviations. Supplementary highly sensible detection methods may be investigated, like digital PCR [78], mass spectrometric based analysis [32], or various sequencing techniques [28].

## Conclusions

We investigated variations in cfDNA concentrations in blood plasma and CSF in a piglet model of perinatal asphyxia. In this proof-of-principle study, methods for cfDNA extraction and assessment were validated and established. We found non-significant tendencies of higher cfDNA levels in piglets exposed to hypoxia compared to controls 30 minutes after the end of hypoxia. Further, cfDNA was higher at post-intervention in the CSF of hypoxic piglets, but the observed differences were not significant. We suggest that an increase in the amount of cfDNA might be an early indicator for stress reactions and perinatal asphyxia damages; however, additional data are required to better understand the biology of cfDNA temporal patterns.

## Supporting information

S1 Fig Standard curve estimations for various sources of DNA. For fluorescent assay for measuring cfDNA a standard curve is required. Several different DNA sources were tested to prepare a standard curve for measuring cfDNA concentrations, including commercial salmon, human, and porcine DNA. The DNA standards were diluted with PBS to the following concentrations: 1250, 750, 500, 250, 125, 100, 75, 50, 25, 12.5, and 6.25 ng/m and determined by the fluorescent assay using SYBR Gold. Briefly, to 10 μl of sample solution an amount of 40 μl of diluted SYBR Gold was added to a final concentration of 1:10.000 and the fluorescence was immediately measured at an emission wavelength of 535 nm and an excitation wavelength of 485 nm using a Victor TM X3 (Perkin Elmer, Waltham, USA). All measurements were performed in parallels.
(DOCX)

S2 Fig Comparison of different fragmentation methods for DNA standards preparation for the qRT-PCR method. The porcine DNA used as standards for the qRT-PCR method with HMBS primers on an Applied Biosystems Viia7 qRT-PCR (Life technologies, Foster City, USA) revealed a larger fragment size and was consequently fragmented prior to the cfDNA assessment. Different fragmentation procedures were examined, including digestion with the restriction enzyme *HhaI* (green), physical fragmentation by UV treatment (red), or without fragmentation (blue). Following fragmentation, the DNA standards were diluted with PBS to the following concentrations: 1250, 750, 500, 250, 125, 100, 75, 50, 25, and 12.5 ng/m (y-axis) versus the Ct value.
(DOCX)

S3 Fig Quantification of cfDNA in blood plasma of a human, adult pig, and newborn piglets. All previous studies on cfDNA are solely based on human samples. In order to get an impression about differences in the amount of cfDNA concentrations in man and piglets, we measured the levels of cfDNA in plasma from a healthy adult male in comparison to the quantity in a healthy adult piglet applying the fluorescence-based method with SYBR Gold. The concentration of cfDNA in ng/ml was determined in duplicates in three independent samples.
(TIF)

S4 Fig Spearman rank-correlation coefficients between the fluorescence assay and the qRT-PCR method at baseline and post-intervention. Scatter plots for the cfDNA blood concentrations of the fluorescence assay versus the qRT-PCR method are shown with regression lines of best fit. The spearman’s rank-correlations were rho = 0.05 at baseline and rho = -0.21 at post-intervention.
(DOCX)

S5 Fig Spearman rank-correlation coefficient between the fluorescence assay and the qRT-PCR method for cfDNA levels in the CSF. A scatter plot for the cfDNA concentrations in the CSF for the fluorescence assay versus the qRT-PCR method is illustrated, including a regression line of best fit. The spearman’s rank-correlation was rho = 0.77.
(DOCX)

S1 File Comparison and evaluation of various methods for the extraction of cfDNA from piglets. (DOCX)
