# Supplementary material for: Temporal patterns of circulating cell-free DNA (cfDNA) in a newborn piglet model of perinatal asphyxia
Source: PLoS One. 2018 Nov 26;13(11):e0206601. doi: 10.1371/journal.pone.0206601 (PMC6261042; doi:10.1371/journal.pone.0206601)
Supplement: S1 Fig — For fluorescent assay for measuring cfDNA a standard curve is required. Several different DNA sources were tested to prepare a standard curve for measuring cfDNA concentrations, including commercial salmon, human, and porcine DNA. The DNA standards were diluted with PBS to the following concentrations: 1250, 750, 500, 250, 125, 100, 75, 50, 25, 12.5, and 6.25 ng/m and determined by the fluorescent assay using SYBR Gold. Briefly, to 10 μl of sample solution an amount of 40 μl of diluted SYBR Gold was added to a final concentration of 1:10.000 and the fluorescence was immediately measured at an emission wavelength of 535 nm and an excitation wavelength of 485 nm using a Victor TM X3 (Perkin Elmer, Waltham, USA). All measurements were performed in parallels. (DOCX) [file pone.0206601.s001.docx]

**Supplementary 2.**
