# Supplementary figures and images for: Temporal patterns of circulating cell-free DNA (cfDNA) in a newborn piglet model of perinatal asphyxia
Source: PLoS One. 2018 Nov 26;13(11):e0206601. doi: 10.1371/journal.pone.0206601 (PMC6261042; doi:10.1371/journal.pone.0206601)

**Supplementary 3.**


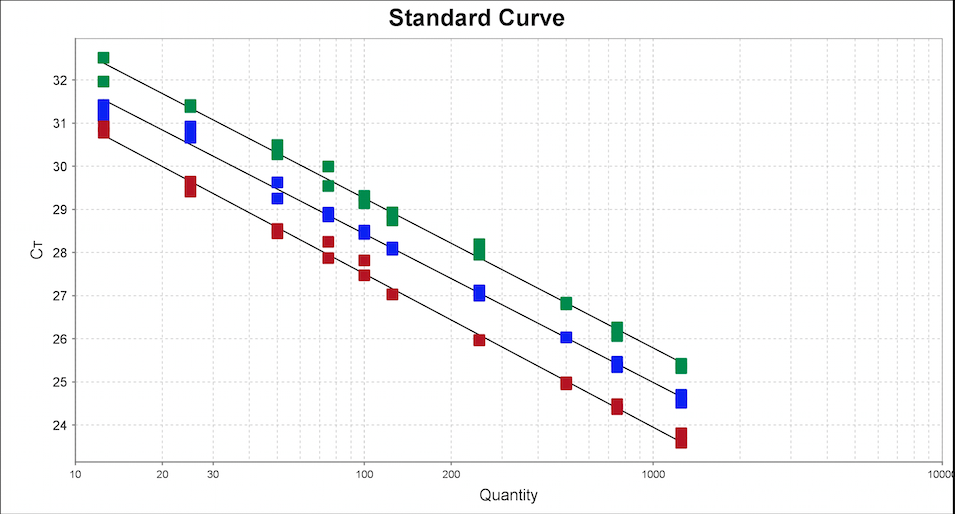

Supplement: S2 Fig — The porcine DNA used as standards for the qRT-PCR method with HMBS primers on an Applied Biosystems Viia7 qRT-PCR (Life technologies, Foster City, USA) revealed a larger fragment size and was consequently fragmented prior to the cfDNA assessment. Different fragmentation procedures were examined, including digestion with the restriction enzyme HhaI (green), physical fragmentation by UV treatment (red), or without fragmentation (blue). Following fragmentation, the DNA standards were diluted with PBS to the following concentrations: 1250, 750, 500, 250, 125, 100, 75, 50, 25, and 12.5 ng/m (y-axis) versus the Ct value. (DOCX) [file pone.0206601.s002.docx]

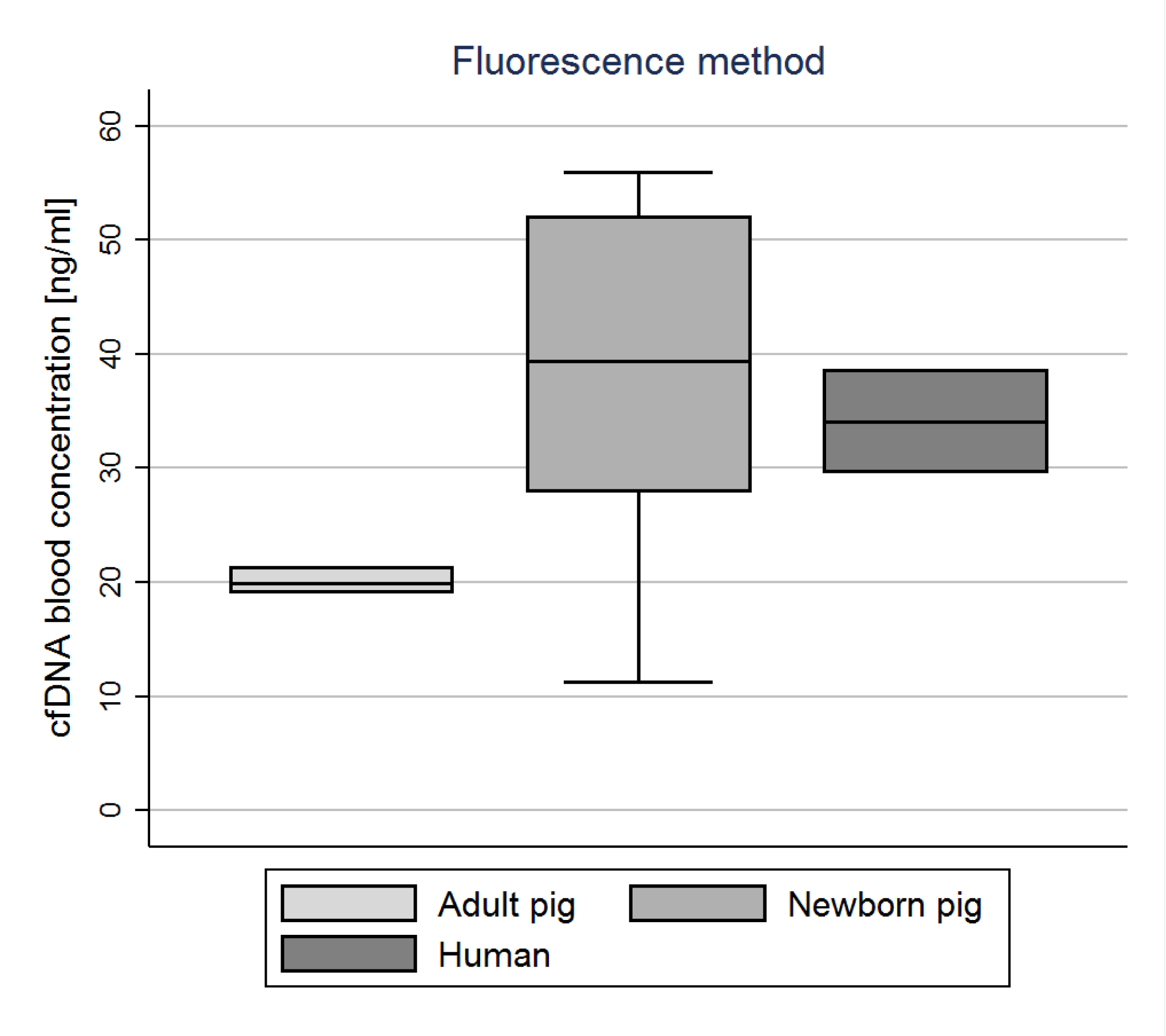

Supplement: S3 Fig — All previous studies on cfDNA are solely based on human samples. In order to get an impression about differences in the amount of cfDNA concentrations in man and piglets, we measured the levels of cfDNA in plasma from a healthy adult male in comparison to the quantity in a healthy adult piglet applying the fluorescence-based method with SYBR Gold. The concentration of cfDNA in ng/ml was determined in duplicates in three independent samples. (TIF) [file pone.0206601.s003.tif]

**Supplementary 4.**


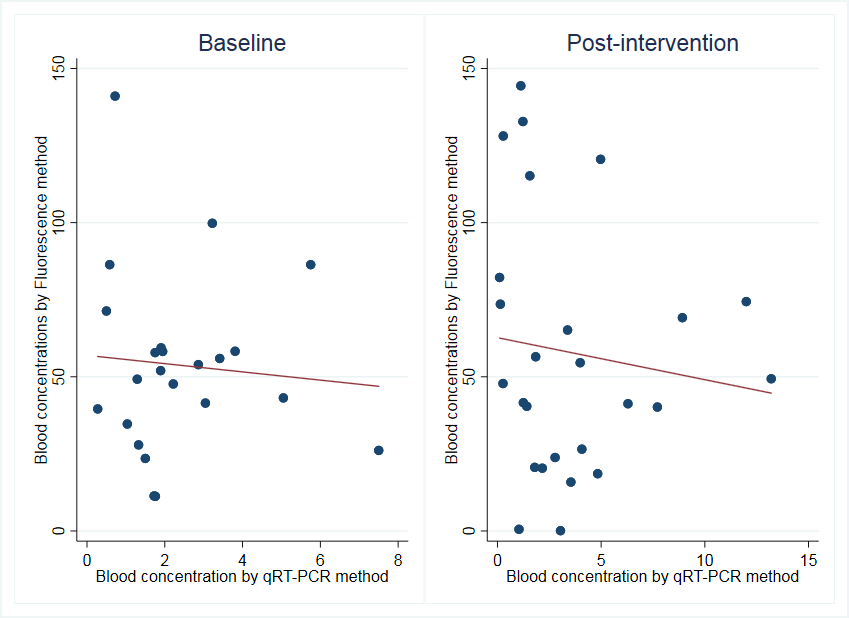

Supplement: S4 Fig — Scatter plots for the cfDNA blood concentrations of the fluorescence assay versus the qRT-PCR method are shown with regression lines of best fit. The spearman’s rank-correlations were rho = 0.05 at baseline and rho = -0.21 at post-intervention. (DOCX) [file pone.0206601.s004.docx]

**Supplementary 5.**


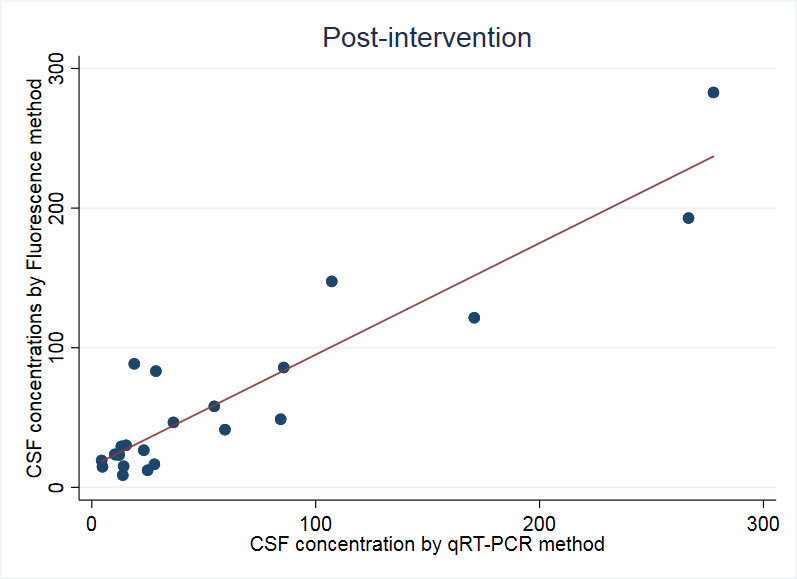

Supplement: S5 Fig — A scatter plot for the cfDNA concentrations in the CSF for the fluorescence assay versus the qRT-PCR method is illustrated, including a regression line of best fit. The spearman’s rank-correlation was rho = 0.77. (DOCX) [file pone.0206601.s005.docx]
