# Supplementary material for: Temporal patterns of circulating cell-free DNA (cfDNA) in a newborn piglet model of perinatal asphyxia
Source: PLoS One. 2018 Nov 26;13(11):e0206601. doi: 10.1371/journal.pone.0206601 (PMC6261042; doi:10.1371/journal.pone.0206601)
Supplement: S1 File — (DOCX) [file pone.0206601.s006.docx]

**Supplementary File 1.**

**Comparison and evaluation of various methods**

**for the extraction of cfDNA from pigs**

For cfDNA extraction, different kits and methods were tested, including DNeasy^®^ Blood & Tissue Kit (DNeasy^®^ kit, Qiagen, Hilden, Germany), NucleoSpin^®^ Plasma XS Kit (NucleoSpin^®^ kit, Macherey-Nagel, Duren, Germany), and Wizard^®^ Genomic DNA Purification Kit (Wizard^®^ kit, Promega, Madison, USA). We also validated other products and kits, including magnetic beads based techniques, kits for isolation of DNA from body fluids, and kits for DNA purification from forensic samples with inconclusive results (data not shown). For all extractions, the recommendations in the manuals of the manufacturers were followed. For the DNeasy^®^ kit, the starting volume was 220 μl of plasma, 20 μl of proteinase K and the DNA was eluted in 200 μl buffer AE. For the NucleoSpin® kit, the start volume was 240 μl and the DNA was eluted with 40 μl elution buffer. In the final step, the samples were incubated at 90°C for 8 minutes to remove the residual ethanol. For the Wizard® kit, 220 μl of plasma samples were used as initial volume and DNA was eluted in 100 μl DNA Rehydration Solution. Three different samples with parallels were analyzed. The handling time was calculated for 20 preps.

The DNeasy^®^ Blood & Tissue Kit from Qiagen, NucleoSpin^®^ Plasma XS Kit from Macherey-Nagel, and Wizard^®^ Genomic DNA Purification Kit from Promega were tested and compared in regards to the handling time, purity, starting volume, and yield (Table 1). Full blood was also investigated with the various kits, resulting in higher DNA concentrations (results not shown); however, rejected due to possible contamination with other cell fragments.

The results showed no differences in yield for the different sample starting volumes of 220 μl or 440 μl and consequently, we used 220 μl for the following experiments.

Although, NucleoSpin^®^ kit resulted in similar yields, the sample was eluted only with 40 μl in opposite to the DNeasy^®^ kit, which was eluted in 200 μl. The DNA purity in an OD ratio of 260/280 was slightly too high in the NucleoSpin^®^ kit (Table 1).

The Wizard^®^ kit turned out to provide high amounts of DNA, it is independent of columns and may be initiated with flexible starting volumes; however, in our hands, the pellet was very difficult to resolve (even after multiple heating) leading to inconsistent yields and poor quality DNA.

The NucleoSpin^®^ kit is especially designed for cfDNA and resulted in high yield of DNA. However, the elution buffer contains ethanol, which we think might be critical for the determination of the final volume and thus the correct DNA concentration, despite the incubation at 90°C for 8 minutes’ step, supposed to remove any residual traces of ethanol.

For the extraction of cfDNA from small plasma samples from pigs, the DNeasy^®^ kit from Qiagen turned out to be the preferred method and accordingly, was used for the following experiments.

We examined the application of the DNeasy^®^ kit in more detail by performing a number of DNA recovery tests to investigate the method-dependent loss of DNA during the extraction. We spiked normal pig plasma samples with 100 ng/μl porcine or human DNA standard. We found a recovery of 30-40% for porcine and 40-50% recovery for human DNA. As a positive control we used DNA digested with DNase resulting in 70-80% DNA degradation.

**Table 1: Comparison of cfDNA extraction efficiency, DNA quality and handling time by three different extraction methods.**

| **Purification kit** | **Producer** | **Extraction method**  **basis** | **Average DNA concentration** | **OD ratio 260/280** | **Handling time (20 preps)** |
| --- | --- | --- | --- | --- | --- |
| DNeasy^®^ Blood & Tissue Kit | Qiagen | Silica membrane binding | 2.35ng/μl | 1.73 | 1.0h |
| NucleoSpin^®^ Plasma XS | Macherey & Nagel | Silica membrane binding | 3.23ng/ μl | 2.64 | 1.5h |
| Wizard^®^ Genomic DNA | Promega | Salt and isopropanol precipitation | 75.06ng/ μl | 0.86 | 2.0h |

We validated three commonly used cfDNA extraction kits, including the DNeasy^®^ Blood & Tissue Kit, the NucleoSpin^®^ Plasma XS Kit, and the Wizard^®^ Genomic DNA Purification Kit for a model of newborn pigs, all methods were fast and easy to handle, but resulted in different yields and purity (Table 1). The DNeasy^®^ and NucleoSpin^®^ kits are similar in the respect that both are based on silica-based membrane-binding spin columns with the ability to bind DNA followed by salt precipitation, in opposite to the Wizard^®^ kit technique, relaying on salt and isopropanol precipitation. In our hands, the DNeasy^®^ kit from Qiagen turned out to be the best-suited method for our purposes, the extraction of cfDNA from small plasma samples from pigs (Table 1). The DNeasy^®^ kit has its advantage being fast and reliable, providing good quality DNA, which has been used for down-stream qRT-PCR in several publications. The disadvantage of the kit was lower yield, restrictions of the starting- and elution volume, and the loss of very small fragments. Another kit from the same producer, the QIAmp^®^ DNA Blood Mini Kit (Qiagen, Hilden, Germany), has also frequently been used for cfDNA studies on human specimen [1-5]. The QIAamp^®^ kit has been validated in comparison to many other DNA isolation methods [6-9]; however, with inconclusive results, exhibiting poor yield [7] or low efficiency [9] to highest efficiency [6] among the primary methods [8]. Functionally, both the DNeasy^®^ kit and the QIAamp^®^ kit isolate DNA from blood samples; however, they differ in that respect that the QIAamp^®^ kit has been designed for human samples, while the protocols for the DNeasy^®^ kit are adapted for non-human samples. The two kits perform more-or-less similarly, and it may not seem significant for most research purposes, still it is important to note that the QC process for the kits are slightly different, e. g. the enzymes used for protein digestion are different between the two kits and there is a distinction for the amount starting material used. We are aware that further methods are available and might be chosen under certain conditions. All DNA extraction methods share their limitation that some fragments might be lost; this may bias the composition of the cfDNA, at least when the concentration is measured by qRT-PCR.

**References**

1. Altimari A, Grigioni AD, Benedettini E, Gabusi E, Schiavina R, Martinelli A, et al. Diagnostic role of circulating free plasma DNA detection in patients with localized prostate cancer. American journal of clinical pathology. 2008;129(5):756-62.

2. Boddy JL, Gal S, Malone PR, Harris AL, Wainscoat JS. Prospective study of quantitation of plasma DNA levels in the diagnosis of malignant versus benign prostate disease. Clin Cancer Res. 2005;11(4):1394-9.

3. Breitbach S, Tug S, Helmig S, Zahn D, Kubiak T, Michal M, et al. Direct quantification of cell-free, circulating DNA from unpurified plasma. PloS one. 2014;9(3):e87838.

4. Catarino R, Ferreira MM, Rodrigues H, Coelho A, Nogal A, Sousa A, et al. Quantification of free circulating tumor DNA as a diagnostic marker for breast cancer. DNA Cell Biol. 2008;27(8):415-21.

5. Mussolin L, Burnelli R, Pillon M, Carraro E, Farruggia P, Todesco A, et al. Plasma cell-free DNA in paediatric lymphomas. Journal of Cancer. 2013;4(4):323-9.

6. Devonshire AS, Whale AS, Gutteridge A, Jones G, Cowen S, Foy CA, et al. Towards standardisation of cell-free DNA measurement in plasma: controls for extraction efficiency, fragment size bias and quantification. Anal Bioanal Chem. 2014;406(26):6499-512.

7. Fleischhacker M, Schmidt B, Weickmann S, Fersching DM, Leszinski GS, Siegele B, et al. Methods for isolation of cell-free plasma DNA strongly affect DNA yield. Clinica chimica acta; international journal of clinical chemistry. 2011;412(23-24):2085-8.

8. Fong SL, Zhang JT, Lim CK, Eu KW, Liu Y. Comparison of 7 methods for extracting cell-free DNA from serum samples of colorectal cancer patients. Clin Chem. 2009;55(3):587-9.

9. Xue X, Teare MD, Holen I, Zhu YM, Woll PJ. Optimizing the yield and utility of circulating cell-free DNA from plasma and serum. Clinica chimica acta; international journal of clinical chemistry. 2009;404(2):100-4.

10. Goldshtein H, Hausmann MJ, Douvdevani A. A rapid direct fluorescent assay for cell-free DNA quantification in biological fluids. Ann Clin Biochem. 2009;46(Pt 6):488-94.
